# Association between Metabolic Disorders and Cholangiocarcinoma: Impact of a Postulated Risk Factor with Rising Incidence

**DOI:** 10.3390/cancers14143483

**Published:** 2022-07-18

**Authors:** Leonardo G. Da Fonseca, Pedro H. Hashizume, Irai Santana de Oliveira, Laura Izquierdo-Sanchez, Lisa Rodrigues da Cunha Saud, Mariana Pinheiro Xerfan, Venancio Avancini Ferreira Alves, Evandro Sobroza de Mello, Paulo Herman, Jesus M. Banales, Claudia P. Oliveira, Flair J. Carrilho

**Affiliations:** 1 Clinical Oncology, Instituto do Cancer do Estado de São Paulo, School of Medicine, University of São Paulo, São Paulo 01246-000, Brazil; pedro.hashizume@gmail.com; 2 São Paulo Clínicas Liver Cancer Group—Instituto do Cancer do Estado de São Paulo, 255 ICHC—9th Floor, Room 9159, São Paulo 05403-000, Brazil; lisasaud@yahoo.com.br (L.R.d.C.S.); venancioavancini@gmail.com (V.A.F.A.); esobroza@usp.br (E.S.d.M.); phernan@uol.com.br (P.H.); cpm@usp.br (C.P.O.); fjcarril@usp.br (F.J.C.); 3 Department of Radiology, Instituto do Cancer do Estado de São Paulo, School of Medicine, University of São Paulo, São Paulo 01246-000, Brazil; irai.oliveira@hc.fm.usp.br; 4 Department of Liver and Gastrointestinal Diseases, Biodonostia Health Research Institute—Donostia University Hospital, University of the Basque Country (UPV/EHU), CIBERehd, Ikerbasque, 48009 San Sebastian, Spain; laura.izquierdo@biodonostia.org (L.I.-S.); jesus.banales@biodonostia.org (J.M.B.); 5 Department of Gastroenterology, Division of Clinical Gastroenterology and Hepatology, School of Medicine, Hospital das Clinicas, University of São Paulo, São Paulo 05403-000, Brazil; mpxerfan@gmail.com; 6 Department of Pathology, University of São Paulo School of Medicine, São Paulo 05403-000, Brazil; 7 Department of Gastroenterology, Division of Digestive Surgery, School of Medicine, Hospital das Clinicas, University of São Paulo, São Paulo 04021-001, Brazil; 8 Department of Biochemistry and Genetics, School of Sciences, University of Navarra, 31080 Pamplona, Spain

**Keywords:** liver cancer, cholangiocarcinoma, metabolic syndrome, diabetes, obesity

## Abstract

**Simple Summary:**

A potential relationship between cholangiocarcinoma and metabolic disorders has been suggested, but there is a lack of published data. This study aimed to describe the prevalence of metabolic disorders in a cohort of 122 patients with cholangiocarcinoma and report clinical outcomes. We found a prevalence of 42.6% of metabolic disorders. There was no significant difference in overall survival between patients with or without metabolic disorders, although there was a better survival in the subgroup of patients undergoing surgical resection. This indicates a need to better explore the association between cholangiocarcinoma in a metabolic background.

**Abstract:**

Introduction and objectives: The incidence of cholangiocarcinoma (CCA) has been increasing globally. Although a concomitant increase in the incidence of metabolic disorders might suggest a causal relationship, the data are scarce. We aimed to describe the prevalence of metabolic disorders in patients with CCA and report the clinical features and outcomes. Patients and Methods: Retrospective study including patients with CCA. Patients were divided into: (1) past history of diabetes or/and overweight/obesity (“metabolic disorder group”) and (2) without any of these features (“non-metabolic-disorder group”). A Cox regression model was used to determine the prognostic factors. Results: 122 patients were included. In total, 36 (29.5%) had overweight/obesity, 24 (19.7%) had diabetes, and 8 (6.6%) had both. A total of 29 (23.8%) patients had resectable disease and received upfront surgery. A total of 104 (85.2%) received chemotherapy for advanced/recurrent disease. The overall survival of the cohort was 14.3 months (95% CI: 10.1–17.3). ECOG-PS 0 (*p* < 0.0001), resectable disease (*p* = 0.018) and absence of vascular invasion (*p* = 0.048) were independently associated with better prognosis. The “metabolic disorder group” (*n* = 52) had a median survival of 15.5 months (95% CI 10.9–33.9) vs. 11.5 months (95% CI 8.4–16.5) in the “non-metabolic-disorder group” (*n* = 70) (HR: 1.10; 95% CI 0.62–1.94). Patients with resectable disease in the “metabolic group” had longer survival than patients in the “non-metabolic group” (43.4 months (95% CI 33.9-NR) vs. 21.8 months (95% CI 8.6–26.9); HR = 0.12, 95% CI 0.03–0.59). Conclusion: Metabolic disorders are frequent among CCA patients. Underlying metabolic comorbidities may be associated with prognosis in resectable CCA. There is a need to explore the mechanism that drives CCA carcinogenesis in a metabolic background.

## 1. Introduction

Cholangiocarcinoma (CAA) represents a heterogeneous entity not only because of the anatomical site of origin (intra-hepatic, perihilar and distal CCA) but also due to differences in molecular features that are being increasingly explored in recent studies [1].

CCA has been associated with several hepatobiliary diseases, such as primary sclerosing cholangitis (PSC), primary biliary cholangitis (PBC), cholelithiasis, viral hepatitis infection, liver fluke infestation, cirrhosis and inflammatory bowel disease. However, these risk factors do not explain either the amount of CCA cases or the increasing incidence. This scenario raises concern about the impact of underexplored factors beyond these etiologies [2].

Non-alcoholic fatty liver disease (NAFLD) is recognized as an emerging risk factor for chronic liver disease, cirrhosis and liver cancer, especially hepatocellular carcinoma (HCC) [3]. It is suggested that NAFLD-related HCC may carry a worse prognosis and poorer response to systemic treatments compared to other etiologies [4]. There have been efforts toward the characterization of patients with metabolic disorders who are at risk of developing cirrhosis and primary liver cancer. Recently, a more practical and inclusive approach suggested the nomenclature of metabolic-associated fatty liver disease (MAFLD) [5]. The MAFLD definition highlights the importance of overweight/obesity and type 2 diabetes mellitus as key additional factors to a steatotic liver. Liver disease related to metabolic factors characterizes the hepatic manifestation of a multisystem disorder, which is heterogeneous in its course and outcomes.

Recent research highlights the role of oxidative stress and lipotoxicity in the progression of liver disease and liver fat deposition [6]. Hyperinsulinemia and insulin resistance, which occur in the context of DM and obesity, are associated with both fat deposition and malignant cell transformation [7]. Insulin is found in bile and stimulates cell proliferation of cholestatic cells, which may promote cholangiocarcinogeneis [6]. Leptin, a hormone secreted by adipous tissue, may play a role in cholangiocytes’ transformation, growth and migration [8]. Therefore, a biological background supports a potential association between metabolic disorders and CCA.

Concomitant increases in the incidence of metabolic disorders and CCA may indicate a causal relationship between these diseases. However, there is a paucity of data supporting this assumption. Considering the potential role of metabolic dysfunction in the carcinogenesis of CCA and its impact on prognosis, the aim of this study is to describe the prevalence of clinical metabolic disorders (such as diabetes and overweight/obesity) in a cohort of patients with CCA and to report the clinical features and outcomes of patients with CCA and metabolic-associated background.

## 2. Materials and Methods

### 2.1. Study Design and Participants

We evaluated a retrospective cohort of patients diagnosed with CCA from October 2013 to January 2021 treated at “Instituto do Cancer do Estado de Sao Paulo” (Brazil). All patients included in this study had confirmed histologic diagnosis of CCA obtained through percutaneous biopsy, fine-needle aspiration or surgical resection. Clinical characteristics, past medical history, underlying liver disease, treatments and outcomes were collected from the medical records. Since the histological classification was significantly modified in the WHO Classification of Tumors in 2019 [9], a histological review as well as assessment of new immunohistochemical markers are under study and will be reported afterward.

Patients were further divided into two groups: (group 1) patients with a past medical history (confirmed or self-reported) of diabetes mellitus (DM) or/and body mass index (BMI) of ≥25 kg/m^2^ and no past history of liver disease, and (group 2) patients without any of the features mentioned. Among the patients in group 1 (“metabolic disorder group”), the imaging features, treatment and outcomes were detailed. The study was approved by the institutional ethics committee (protocol number 3.807.496).

### 2.2. Management and Treatment Protocol

According to the local protocol, all patients referred to our institution have CCA diagnosis confirmed by reassessment of the external tissue sample or newly obtained samples. Baseline evaluation consists of performance status assessment using the Eastern Cooperative Oncology group (ECOG) scale, past medical history, comorbidities, liver function tests and general laboratory parameters. Radiological studies are performed to assess the loco-regional or distant spread, staging and resectability. Imaging consists of chest, abdomen and pelvis computed tomography (CT) scans. Whenever required, liver magnetic resonance imaging (MRI) or cholangiopancreatography are performed. Serum tumor markers, such as carbohydrate antigen (CA) 19–9 and carcinoembryonic antigen (CEA), are routinely collected at the baseline assessment and periodically during the treatment and follow-up.

Treatments are provided according to the local protocol, which is in line with the main guidelines adopted globally [10]. Briefly, the treatment strategy varies for each type of CCA depending on its site of origin. Patients with local disease who are potential candidates for resections are usually discussed in weekly multidisciplinary tumor boards for surgery indication. After resection, no adjuvant treatment is routinely offered, and patients are followed with imaging assessment every 6 months. Patients with locally advanced/unresectable disease and those who have distant metastasis are considered for systemic treatment. Patients with ECOG performance status of 0–2 with no organ dysfunctions are suitable for first-line chemotherapy with cisplatin-gemcitabine combination, according to the ABC-02 trial [11]. Alternatively, some patients may start gemcitabine monotherapy or other regimens (gemcitabine-oxaliplatin or fluoropyrimidine-oxaliplatin) as first-line treatment at the physician’s discretion. After progression to first-line therapy, patients who are fit for subsequent treatment are often considered for receiving 5-fluorouracil-based regimens as a second-line treatment. Whenever required, candidates for systemic treatment receive biliary drainage.

### 2.3. Patients with a Background of Metabolic Disorders

Patients with a past medical history of DM and/or BMI of ≥25 kg/m^2^ were selected from the total cohort and grouped as a “metabolic disorder” group. Clinical characteristics and outcomes of this specific group were analyzed. Baseline images were also evaluated in order to assess the prevalence of steatosis. The manual mean liver attenuation was measured in Hounsfield units (HU) by using a simple and previously validated technique, which consists of the placement of a ROI over a representative parenchymal portion of the right hepatic lobe. The criteria used for defining steatosis were liver attenuation ≤ 40 HU on unenhanced CT images. Although many criteria have been previously used to determine liver steatosis with variable sensitivity and specificity, it is suggested that a liver attenuation value ≤ 40 HU represents the most accurate criterion for detecting moderate-to-severe disease [12,13].

### 2.4. Statistical Analysis

Descriptive methods were used to analyze the incidence of risk factors in the total cohort and to report baseline and demographic features. Continuous variables were expressed as mean, median, ranges or interquartile ranges (IQR). Categorical variables were expressed as frequency. Comparisons between the group of interest (“metabolic disorder group”) and the group of patients with no background of metabolic disorders were performed. Categorical variables were compared using the χ^2^-test or Fischer’s exact test when appropriate. Continuous variables were compared using Student’s *t*-test. Overall survival (OS) was estimated using the Kaplan–Meier method, and curves were compared by using log-rank. For the analysis including the whole cohort, a Cox regression model, including variables that showed significance in the univariate analysis, was performed to evaluate the independent prognostic factors and calculate the hazard ratios (HR) and 95% confidence intervals (CI). For time-to-event analysis including only patients submitted for surgery (recurrence-free survival and overall survival from surgery), a Cox regression model was performed, including variables associated with outcomes in patients with CCA submitted for resection [14,15,16]: primary site (intrahepatic, perihilar or distal), vascular invasion (yes or no), status of resection margin (R0, R1 or R2), baseline CA 19.9 (< or ≥150 U/mL), nodal disease (N1 or N0), number of nodules (uni- or multinodular) and ECOG-PS (0, 1 and ≥2). A *p* < 0.05 was considered significant. Data were evaluated using the STATA software version 15.0.

## 3. Results

### 3.1. Baseline Characteristics

From October 2013 to January 2021, 122 patients with confirmed diagnosis of CCA were included in the present analysis. The median age was 62 years (IQR 55–67), 72 (59%) were female, and the predominant primary site was intrahepatic CCA (*n* = 48; 39%) followed by distal CCA (*n* = 41, 33.6%) and perihilar CCA (*n* = 30, 24.6%). History of alcohol consumption was reported by 20 (16.4%) patients and smoking by 54 (44.3%). Regarding metabolic factors, the median BMI was 23 kg/m^2^ [IQR 20–26], 23 (18.9%) patients had BMI 25–30 kg/m^2^, 13 (10.7%) patients had BMI ≥ 30 kg/m^2^, 24 (19.7%) patients had DM, and 8 (6.6%) patients had both DM and BMI ≥ 25 kg/m^2^. The other risk factors found were PSC in two (1.7%) patients, cirrhosis in one (0.8%), viral hepatitis in four (3.2%) and human immunodeficiency virus (HIV) infection in two (1.6%) patients. The majority of patients had a performance status of 0–1 (*n* = 84; 68.9%), and 65 (53.8%) had metastatic/unresectable disease at diagnosis. Median gamma-glutamyl transferase (GGT = 423 U/L) and alkaline phosphates (ALP = 272 U/L) were higher than the upper limit of the reference range (30 U/L and 150 U/L, respectively), while the other median laboratory parameters were within the normal range or only slightly altered.

Regarding patients with DM, 23 patients were classified as having type II DM, and 1 patient had type I DM. The median time from DM diagnosis to CCA diagnosis was 6 years (IQR: 2.5–12.2 years), and all patients reported regular follow-up since diagnosis. At CCA diagnosis, 18 (75%) patients were using metformin, 9 (37.5%) patients were using glicazide, 4 (16.7%) were using glibenclamide, and 3 patients were using dapaglifozin (12.5%). Eleven (45.8%) patients reported routine use of insulin, while three (12.5%) patients reported previous use before CCA diagnosis. More than one anti-DM drug was required for 15 (62.5%) patients, and 2 (8.3%) patients reported DM control with dietary and lifestyle habits without current medication. Baseline hemoglobin A1C (within 3 months of CCA diagnosis) was available for 19 (79.1%) patients, with a median of 7.3% (IQR, 6.41–8.24%). In the non-metabolic-disorder group, 17 (24.3%) patients had available hemoglobin A1C with a median of 5.3 (IQR 5.1–5.6).

Regarding the therapeutic strategy, 29 (23.8%) patients were treated with upfront curative-intent resection. Chemotherapy was delivered to 104 patients, given that cisplatin-gemcitabine was the most used regimen (*n* = 98; 94.2%) followed by gemcitabine-oxaliplatin (*n* = 3; 2.9%), gemcitabine monotherapy (*n* = 2; 1.9%) and mFLOX (*n* = 1; 0.9%). Biliary drainage was required in 19 (15.5%) and stenting in 66 (54.1%) patients. Table 1 shows the baseline characteristics of the entire cohort and subgroups.

### 3.2. Comparison between Groups According to Metabolic Factors

The group of patients with metabolic factors (“metabolic disorder group”) accounted for 52 (42.6%) patients, while the other 70 (57.4%) patients represented the “non-metabolic group”. As expected, the metabolic group had higher median weight (67.5 vs. 55.0 kg; *p* < 0.0001) and higher median BMI (26.0 vs. 21.0 kg/m^2^, *p* < 0.0001). Moreover, the metabolic group had a higher prevalence of women (69.2 vs. 36%; *p* = 0.048) and non-smokers (75% vs. 41.4%; *p* < 0.0001).

On the other hand, there was no difference regarding prognostic factors, such as ECOG-PS, metastatic disease, positive lymph node, primary site and choice of therapeutic strategies, between “metabolic disorder” and “non-metabolic-disorder” groups. (Table 1).

The median liver attenuation in the “metabolic disorder group” was 56 UH (IQR: 46.2–56.2). Only one patient in the “metabolic disorder group” met the radiologic criteria for hepatic steatosis, with a liver attenuation of 36 UH.

### 3.3. Clinical Outcomes and Overall Survival

**Figure 1:**
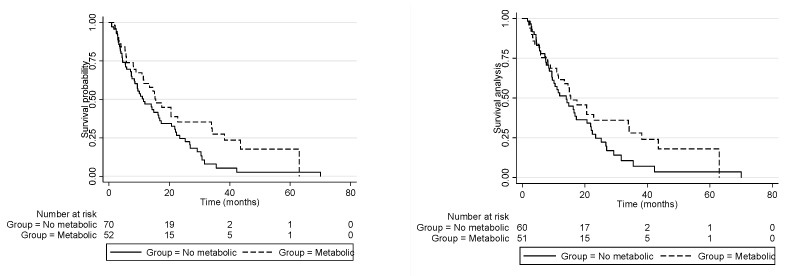
Kaplan–Meier curves showing: (**Left**) overall survival according to subgroup with metabolic disorders vs. no metabolic disorders. The median overall survival of the “metabolic disorder” group was 15.5 months (95% CI 10.9–33.9), and the median OS of the “non-metabolic-disorder” group was 11.5 months (95% CI 8.4–16.5), adjusted HR: 1.09 (0.62–1.94), *p* = 0.745. (**Right**) overall survival according to subgroup with metabolic disorders vs. no metabolic disorders, excluding patients managed with best supportive care. The median overall survival of the “metabolic disorder” group was 15.5 months (95% CI 11.4–33.9), and the median OS of the “non-metabolic-disorder” group was 14.0 months (95% CI 9.4–17.3), adjusted HR: 1.32 (0.74–2.35), *p* = 0.351.

The median OS of the “metabolic disorder” group was 15.5 months (95% CI 10.9–33.9) compared to 11.5 months for the “non-metabolic-disorder” group (95% CI 8.4–16.5; univariate *p* = 0.048). In the univariate analysis, other variables were associated with better OS: CA19.9 < 150 U/mL (*p* = 0.0003); diabetes mellitus (*p* = 0.04); ECOG-PS 0 (*p* < 0.001); absence of metastasis (*p* = 0.0012); absence of vascular invasion (*p* = 0.001); and upfront resection (*p* < 0.001). In the multivariate analysis, ECOG-PS (*p* < 0.0001), surgery (*p* = 0.018) and absence of vascular invasion (*p* = 0.048) were independently associated with survival, while there was no significant survival difference between the “metabolic disorder” and “non-metabolic-disorder” groups in the multivariate analysis (adjusted HR: 1.09; 95% CI: 0.62–1.94). (Table 2 and Figure 1).

There was no correlation between the group (metabolic vs. non-metabolic) and the tumor site (χ^2^ = 1.46; *p* = 0.691). Interestingly, the median overall survival for patients with intrahepatic cholangiocarcinoma and in the metabolic group (*n* = 30) was 7.7 months (95% CI 3.1–11.9), and for patients with intrahepatic cholangiocarcinoma and in the “non-metabolic” group (*n* = 18), it was 15.5 months (95% CI 8.2—not reached) (univariate *p* = 0.006). However, after performing a Cox regression model including only patients with intrahepatic cholangiocarcinoma, the metabolic group was not associated with prognosis (HR = 0.43, 95% CI 0.13–1.42).

### 3.4. Treatment Strategies and Clinical Outcomes

**Figure 2:**
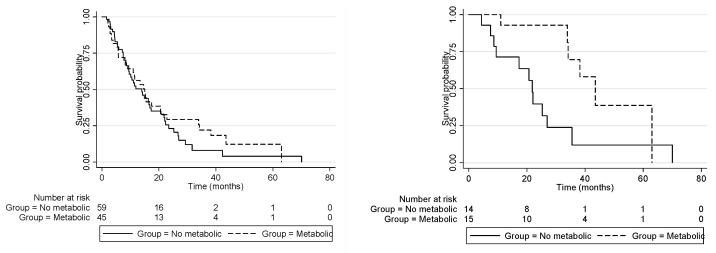
Overall survival. (**Left**) Patients treated with chemotherapy: the median OS was 11.5 months (95% CI 8.4–14.8), and there was no difference between metabolic and non-metabolic groups (11.5 months [95% CI 5.8–15.3] vs. 11.5 months [95% CI 6.7–16.2]; *p* = 0.406). (**Right**) Patients treated with surgery: the median of the metabolic group was significantly better than that of the non-metabolic group (43.4 [95% CI 33.9-NR] vs. 21.8 months [95% CI 8.6–26.9]; HR = 0.23, 95% CI 0.06–0.86; *p* = 0.029.

Regarding patients who were submitted for upfront surgery (*n* = 29), 21 (75%) presented disease recurrence with a median time to recurrence of 14 months (95% CI 8.2–19.8). Patients in the “metabolic disorder” group (*n* = 8) had significantly longer relapse-free survival (15.6 months, 95% CI 8.2–24.6) compared to patients in the “non-metabolic group” (13.5 months, 95% CI 3.5–19.8, multivariate *p* = 0.02). Accordingly, the median overall survival of the metabolic group after resection was significantly higher than the non-metabolic group (43.4 [95% CI 33.9-NR] vs. 21.8 months [95% CI 8.6–26.9]; adjusted HR = 0.23, 95% CI 0.06–0.86; *p* = 0.029) (Figure 2).

Considering the patients who were treated with chemotherapy, the median OS was 11.5 months (95% CI 8.4–14.8), and there was no difference between the metabolic and non-metabolic groups (11.5 months [95% CI 5.8–15.3] vs. 11.5 months [95% CI 6.7–16.2]) with an adjusted HR = 0.82 (95% CI 0.52–1.30; *p* = 0.406).

We observed a higher rate of patients managed with best supportive care in the non-metabolic-disorder group (14.3% vs. 1%, *p* = 0.018). Therefore, we also performed a survival analysis excluding these patients. After excluding patients managed with best supportive care, the median OS for the non-metabolic group (*n* = 60) was 14.01 months (95% CI 9.42–17.29) vs. 15.47 (95% CI 11.40–33.92) for the metabolic group (*n* = 51); HR: 1.32 (95% CI 0.74–2.35), *p* = 0.351 Figure 1. Accordingly, we also performed a Cox regression model excluding patients treated with best supportive care and observed that the same variables remained independently associated with survival (ECOG-PS: *p* = 0.017; surgery: *p* = 0.03; and absence of vascular invasion: *p* = 0.032).

Detailed results regarding OS, progression-free survival and response rate are shown in Table 3.

## 4. Discussion

Our study demonstrates that metabolic-associated factors coexist in a significant proportion of patients with CCA, denoting a potential role of these factors in CCA initiation and progression. Although metabolic factors were not clearly associated with prognosis in the whole cohort, the subgroup of patients with metabolic-associated factors submitted for surgical resection had better prognosis compared to patients with no metabolic comorbidities submitted for surgery.

The relationship between HCC and NAFLD/MAFLD is well described, and this is projected to be the prevailing etiology in the upcoming years across Western countries. However, data related to CCA and metabolic disorders are scarce and contrasting. In the past years, few epidemiologic studies addressed this topic [17,18,19]. A Danish national registry demonstrated that diabetes was associated with an increased risk of intrahepatic CCA, while obesity was unrelated [18]. Other analysis from the Surveillance, Epidemiology and End Results (SEER) reported an association between intrahepatic CCA, but not extrahepatic CCA, with obesity [17]. In a case–control study using a UK primary care database, both obesity and diabetes were significantly related to CCA incidence. Beyond Western reports, a case–control study in China also showed a positive correlation between CCA and the metabolic syndrome, and obesity was associated with intra- and extra-hepatic CCA, while diabetes was only related to intra-hepatic CCA [20].

Although our study was not designed to be a case–control analysis, the high prevalence of diabetes and overweight/obesity in the present cohort compared to other well-known risk factors (such as PSC, viral hepatitis and cirrhosis) strongly suggests that the association of these factors with CCA is also relevant in our population. Smoking, another possible risk factor for CCA [21], was less prevalent among patients with metabolic disorders. It is increasingly evident that intra- and extrahepatic CCAs are associated with distinct mutational profile, [1], while both were associated with high prevalence of metabolic factors in the present cohort. This suggests that metabolic disorders might play an independent role in the biliary tract carcinogenesis beyond the genetic alterations.

The mechanisms that drive cholangiocarcinogenesis in patients with metabolic-associated factors are not completely understood. DM increases the risk of several types of cancers; therefore, the mechanisms may also be applied to CCA. The available data suggest a mitogenic effect of insulin, hyperactivation of insulin growth factor receptor and an enhancing activity of Wnt/B-catenin signaling and activated transcripts under supra-physiological levels of glucose [22,23]. Glucose metabolism in tumor cells is also reported to regulate local tumor immunity [24]. Leptin, as well as other pro-inflammatory cytokines, such as interlukin-6 and tumor necrosis factor, are increased in obesity and may be linked to cholangiocarcinogenesis because cholangiocytes express their receptors [8].

A large proportion of cases in the present cohort were diagnosed at unresectable/advanced stages, and the management was mainly based on cisplatin-gemcitabine combination, according to international guidelines. The outcome of patients treated with chemotherapy was comparable to what is reported in large phase III trials or international registries [25,26,27], with a median overall survival of less than 1 year. In the present analysis, metabolic disorders did not affect prognosis among patients treated with chemotherapy for advanced CCA. On the contrary, when analyzing only patients who were submitted for upfront curative surgery, we found significantly better outcomes in those patients with metabolic risk factors, after adjusting for lymph node involvement, resection margin and the level of preoperative CA 19.9. Although the sample size was small, these findings generate the hypothesis that background etiology may impact the risk of recurrence and mortality after resection. Similar findings have been demonstrated in resected MAFLD-HCC in a recently published metanalysis [28,29]. In our case, a potential explanation is that patients tend to be more adherent to medical treatment and lifestyle changes after the diagnosis of CCA, and it would minimize the carcinogenic effect of overweight and altered glucose metabolism in the follow-up. In this regard, the published data have shown that biguanides, such as metformin, promote a 60% reduction in the risk of developing iCCA [19], while aspirin and statins were shown to reduce the risk of death among patients with CCA [30,31].

There are controversial data regarding the impact of metabolic risk factors and outcomes in patients with CCA. In a database from Mayo Clinic Florida, BMI did not make a significant impact on the survival, and there was no difference in the overall survival for obese compared to normal or overweight patients with CCA [32]. Nevertheless, a multicenter study suggested that NASH was related to higher mortality rate in patients with CCA [33].

We observed a higher rate of patients managed with best supportive care in the non-metabolic-disorder group (14.3 vs. 1%, *p* = 0.018). The reason for that seems to be related to the fact that patients who are managed with best supportive care tend to present with poor health condition, weight loss and cachexia. Therefore, they are less likely to meet the criteria for a metabolic disorder. Additionally, as these patients usually present with uncontrolled symptoms, no further investigations are performed once the aim of the treatment is to exclusively provide symptom control and supportive care. Even so, this imbalance did not affect the survival analysis and conclusion, as shown in the results.

We found a non-statistically significant trend toward a better survival for patients in the non-metabolic group with intrahepatic CCA. The small sample size prevents us from drawing any conclusion, but further studies might explore the impact of metabolic disorders in different biliary tumor sites.

Our study has certain limitations. First, its single-center, retrospective and uncontrolled design precludes definitive conclusions but supports hypotheses for further studies. Second, due to the retrospective nature of the study, detailed information on risk factors, diagnosis, management and follow-up may be missing. However, it is a pioneer study in the description of this relationship (metabolic disease and CCA), including patients from Latin America (Brazil). An initial report from a Latin American multicenter registry suggested that the most frequent risk factors were overweight/obesity, diabetes and NAFLD [34]. A more comprehensive analysis on a potential prognostic association in a regional multicenter registry is warranted, as risk factors may vary across different regions. External data suggest that NASH-related intrahepatic CCA carries worse prognosis after resection, while NAFLD-related intrahepatic CCA does not [33]. Once we found controversial prognostic impact of metabolic disorders in a real-world cohort selected according to clinical features (and not histological confirmation of NASH), there is a need to further validate the hypothesis that patients with a metabolic background present a better prognosis after resection in a prospective cohort with confirmed risk factors and a long-term follow-up.

In summary, metabolic disorders, such overweight/obesity and diabetes, arise as one major global health issue, and its relationship with biliary tract cancers, although attributable, is not totally understood. Our data call the attention for a remarkable prevalence of metabolic factors among CCA patients and the need to further investigate patients with metabolic disorders with suspected laboratorial liver alterations. The prognostic impact of a metabolic background on CCA is controversial, and it may vary according to the tumor site and stage. The comprehension of its role in pathophysiology and molecular alterations, and moreover, in prognostic and therapeutic implications, warrants future investigation.

## Figures and Tables

**Table 1:** Baseline features of the total cohort and subgroups according to the coexistence of metabolic disorders.

Variables	Total	Metabolic Disorder Group	Non-Metabolic-Disorder Group	*p* Value
*n* (%)	122	52 (42.6%)	70 (57.4%)	
Median age, years (IQR)	62 (55–67)	64 (56–68.5)	59 (53–64)	0.199
Gender				0.048
Woman, *n* (%)	72 (59.0%)	36 (69.2%)	36 (51.4%)	
Man, *n* (%)	50 (40.9%)	16 (30.8%)	34 (48.6%)	
Conditions				
Weight, kilograms (IQR)	59 (51.7–68)	67.5 (59.9–79)	55 (48–59)	<0.0001
Height, centimeters (IQR)	159 (153–166)	157.5 (152.5–164.5)	160 (154–168)	0.1991
BMI, kg/m^2^ (IQR)	23 (20–26)	26 (24.5–29.5)	21 (18–23)	<0.0001
Smoking, *n* (%)	54 (44.3%)	13 (25%)	41 (58.6%)	<0.0001
Alcohol, *n* (%)	20 (16.4%)	6 (11.6%)	14 (20%)	0.212
PSC, *n* (%)	2 (1.7%)	0 (0%)	2 (2.9%)	0.219
Cholelithiasis, *n* (%)	11 (9.0%)	4 (7.7%)	7 (10%)	0.354
Cirrhosis, *n* (%)	1 (0.8%)	0 (0%)	1 (1.4%)	0.387
Viral hepatitis	4 (3.2%)	2 (3.8%)	2 (2.9%)	0.742
HIV	2 (1.6%)	0 (0%)	2 (2.9%)	0.219
Primary site				0.691
Intrahepatic	48 (39.3%)	18 (34.6%)	30 (42.9%)	
Perihilar	30 (24.6%)	13 (25%)	17 (24.3%)	
Distal	41 (33.6%)	19 (36.5%)	22 (31.4%)	
Undefined	3 (2.5%)	2 (3.9%)	1 (1.4%)	
Performance status				0.636
0–1, *n* (%)	84 (68.9)	37 (71.1%)	47 (67.1%)	
2–4, *n* (%)	38 (31.1%)	15 (28.9%)	23 (32.9%)	
Number of liver nodules				0.124
Unicentric, *n* (%)	73 (59.9%)	37 (71.2%)	36 (51.4%)	
Multicentric, *n* (%)	44 (36.1%)	13 (25%)	31 (44.3%)	
Non-Applicable, *n* (%)	5 (4.1%)	2 (3.9%)	3 (4.3%)	
Vascular invasion, *n* (%)	44 (36.1%)	17 (32.7%)	27 (38.6%)	0.576
Clinical positive node, *n* (%)	55 (45.1%)	26 (50%)	29 (41.4%)	0.178
Distant metastasis, *n* (%)	61 (50%)	25 (48.1%)	36 (51.4%)	0.595
Tumor status (pT and/or cT)				0.100
T1, *n* (%)	9 (7.4%)	7 (13.5%)	2 (2.9%)	
T2, *n* (%)	39 (32.0%)	21 (40.4%)	18 (25.7%)	
T3, *n* (%)	42 (34.4%)	14 (26.9%)	28 (40%)	
T4, *n* (%)	25 (20.4%)	7 (13.5%)	18 (25.7%)	
Tx, *n* (%)	7 (5.7%)	3 (5.8%)	4 (5.7%)	
Nodal status (pN or cN)				0.541
N0, *n* (%)	51 (41.8%)	18 (34.6%)	33 (47.1%)	
N1, *n* (%)	53 (43.4%)	24 (46.2%)	29 (41.4%)	
N2, *n* (%)	4 (3.3%)	3 (5.8%)	1 (1.4%)	
Nx, *n* (%)	14 (11.5%)	7 (13.5%)	7 (10%)	
Metastasis (cM and/or pM)				0.768
M0, *n* (%)	57 (46.7%)	27 (51.9%)	30 (42.9%)	
M1, *n* (%)	65 (53.8%)	25 (48.1%)	40 (57.1%)	
Growth pattern				0.174
Mass Forming, *n* (%)	72 (59.0%)	27 (51.9%)	45 (64.3%)	
Periductal infiltrating, *n* (%)	23 (18.9%)	10 (19.2%)	13 (18.6%)	
Intraductular growth, *n* (%)	6 (4.9%)	1 (1.9%)	5 (7.1%)	
Not available, *n* (%)	22 (18.0%)	15 (28.5%)	7 (10.0%)	
Laboratory				
AST, U/L median (IQR)	44.5 (29–76)	43.5 (24.7–57.2)	46 (32–75.5)	0.848
ALT, U/L median (IQR)	44 (30–68)	45 (29–74)	44.5 (30–76.8)	0.202
ALP, U/L median (IQR)	272 (141–569)	226 (143–429)	289 (134.5–588)	0.189
GGT, U/L median (IQR)	423 (158–752)	413 (132.7–630.5)	423 (165–793)	0.098
Bilirubin, mg/dL median (IQR)	1.03 (0.45–4.50)	0.98 (0.43–4.01)	1.25 (0.45–4.48)	0.707
Cholesterol, mg/dL median (IQR)	148 (132–201)	201 (181–213)	141 (130–143.5)	0.404
LDL, mg/dL median (IQR)	93 (94–129)	127 (109–140)	87 (74–91)	0.383
TG mg/dL, median (IQR)	118.5 (92–188)	132 (112–199)	92 (71–114)	0.777
AFP, ng/mL median (IQR)	3.1 (2–5.7)	3 (2.2–4.0)	3.35 (2.05–5.37)	0.157
CEA, ng/L median (IQR)	3.61 (2.08–14.5)	3.62 (2.05–12.87)	3.65 (2.1–16.87)	0.223
CA19.9, ng/L median (IQR)	145 (35.6–614)	112.45 (38.82–461.75)	151.85 (26.64–675.2)	0.855
Glucose, mg/dL median (IQR)	104 (100–106)	111 (98.5–125)	95 (83–107.5)	0.542
Albumin, g/dL median (IQR)	3.6 (3.3–4.1)	3.5 (3.1–4.1)	3.7 (3.3–4.1)	0.164
Neutrophils, /mm^3^ median (IQR)	6620 (5800–7400)	6600 (5800–7400)	6700 (5700–7405)	0.621
Lymphocytes, /mm^3^ median (IQR)	2100 (1400–2950)	2300 (1500–3020)	1900 (1395–2950)	0.701
Hemoglobin, g/dL median (IQR)	11.9 (10.8–12.9)	12.05 (11.0–13.07)	11.6 (10.4–12.7)	0.456
Platelets, 10^3^ median (IQR)	270 (201–351)	279 (185–347)	266 (212–354)	0.173
INR, median (IQR)	1.15 (1.05–1.26)	1.13 (1.05–1.24)	1.16 (1.05–1.28)	0.576
Therapeutic management				
Curative intent resection, *n* (%)	29 (23.8%)	15 (28.8%)	14 (20%)	0.256
Recurrence after surgery, *n* (%)	21 (17.2%)	8 (15.4%)	13 (18.6%)	0.039
Chemotherapy, *n* (%)	104 (85.2%)	45 (76.9%)	59 (84.3%)	0.256
Best supportive care, *n* (%)	11 (9%)	1 (1.9%)	10 (14.3%)	0.018
Biliary stent, *n* (%)	66 (54.1%)	25 (48.1%)	41 (58.6%)	0.25
Biliary drainage, *n* (%)	19 (15.5%)	9 (17.3%)	10 (14.3%)	0.328

IQR: interquartile range; BMI: body mass index; PSC: Primary Sclerosing cholangitis; HIV: human deficiency virus; AST: aspartate aminotransferase; ALT: alanine aminotransferase; ALP: alkaline phosphatase; GGT: gamma-glutamyl transferase; Bili: bilirubin; LDL: low-density lipoprotein; TG: triglycerides; AFP: alpha-fetoprotein; CEA: carcinoembrionic antigen; INR: international normalized ratio; U: unit; ng: nanograms; L: liter; dL: deciliter; mg: milligrams.

**Table 2:** Survival by subgroups and uni- and multivariate analysis.

Subgroups (*n*)	*n*	Median Overall Survival (95% CI)	Univariate *p*	HR (95% CI) Multivariate, *p* Value
Man	50	11.5 (8.6–20.6)	0.88	
Woman	72	14.6 (9.4–17.4)		
Obesity	13	15.5 (5.8–NR)	0.77	
No obesity	109	13.4 (9.6–17.3)		
Diabetes	24	22.8 (5.8–38.2)	0.04	0.54 (0.25–1.15), *p* = 0.111
No diabetes	98	11.9 (9.4–15.5)		
Smoking	54	10.1 (7.4–15.3)	0.17	
No smoking	68	16.5 (11.1–21.8)		
Alcohol	20	16.2 (5.7–22.4)	0.93	
No alcohol	102	14.0 (9.6–17.3)		
Undefined primary site	3	3.5 (3.5–NR)	0.539	
Distal	41	20.6 (14.8–25.3)		
Intrahepatic	48	10.5 (7.4–14.6)		
Perihilar	30	8.9 (5.4–26.7)		
PS0	42	16.8 (14.2–29.2)	<0.001	1.86 (1.43–2.41), *p* < 0.0001
PS1	42	13.4 (9.4–20.6)		
PS2	25	11.1 (5.5–22.0)		
PS3	12	3.0 (2.1–5.4)		
PS4	1	NR (NR–NR)		
Vascular invasion	44	5.9 (4.5–11.4)	0.0012	1.61 (1.01–2.58), *p* = 0.048
No vascular invasion	74	16.5 (11.9–22.4)		
Node positive	55	8.9 (5.8–15.3)	0.14	
Node negative	62	16.5 (10.9–26.9)		
Metastasis	61	8.9 (5.4–14.0)	0.001	1.47 (0.91–2.41), *p* = 0.118
No metastasis	59	21.8 (13.4–26.9)		
Biliary stent	66	13.4 (7.2–17.3)	0.07	
No biliary stent	56	15.5 (9.6–25.3)		
Resection	29	34.2 (22.0–43.5)	<0.0001	0.42 (0.21–0.86), *p* = 0.018
No resection	93	10.5 (7.5–14.0)		
Family history of neoplasia	50	20.7 (11.9–25.2)	0.11	
No family history of neoplasia	67	11.5 (7.7–16.2)		
CA19.9 < 150 U/mL	64	17.5 (11.5–22.4)	0.0003	1.47 (0.91–2.36), *p* = 0.112
CA19.9 ≥ 150 U/mL	58	8.1 (5.7–14.3)		
Metabolic disorder group	52	15.5 (10.9–33.9)	0.048	1.09 (0.62–1.94), *p* = 0.745
Non-metabolic-disorder group	70	11.5 (8.4–16.5)		

PS: performance status; HR: Hazard ratio; CI: confidence interval; NR: not-reached; U/mL: Unit/milliliter.

**Table 3:** Overall survival, progression-free survival and relapse-free survival in the total cohort and subgroups.

**Overall Survival**	***n***	**Median (95% CI)**
Total cohort, months (95% CI)	122	14.3 months (10.1–17.3)
Treated with surgery, months (95% CI)	8	35.5 months (35–NR)
Treated with surgery plus CT at recurrence, months (95% CI)	21	26.9 months (17.3–43.5)
Treated with chemotherapy only, months (95% CI)	82	11.5 months (8.4–14.8)
Best supportive care only, months (95% CI)	11	3.5 months (0.2–5.3)
“Non-metabolic group” treated with surgery, months (95% CI)	14	21.8 months (95% CI 8.6–26.9)
“Metabolic group” treated with surgery, months (95% CI)	15	43.4 months (95% CI 33.9–NR)
“Non-metabolic group” treated with chemotherapy, months (95% CI)	46	11.5 months (95% CI 6.7–16.2)
“Metabolic group” treated with chemotherapy, months (95% CI)	36	11.5 months (95% CI 5.8–15.3)
**Progression-Free Survival (PFS)**	***n***	**Median (95% CI)**
PFS to first-line chemotherapy, months (95% CI)	82	4.3 months (3.5–5.1)
PFS to first-line chemotherapy “metabolic group”, months (95% CI)	36	5.8 months (3.8–8)
PFS to first-line chemotherapy “non-metabolic group”, months (95% CI)	46	4.2 months (2.6–4.7)
**Recurrence-Free Survival after Surgery (RFS)**	***n***	**Median (95% CI)**
RFS after surgery, months (95% CI)	21	14 months (8.2–19.8)
RFS after surgery “metabolic group”, months (95% CI)	8	15.6 months (8.2–24.6)
RFS after surgery “non-metabolic group”, months (95% CI)	13	13.5 months (3.5–19.8)
**Survival according to Radiologic Response**	***n***	**Median (95% CI)**
Partial response, months (95% CI)	28	17.4 months (14.6–38.21)
Stable disease, months (95% CI)	39	20.5 months (11.9–22.8)
Progressive disease, months (95% CI)	30	7.4 months (5.4–9.6)
No assessment, months (95% CI)	7	2.8 months (1.6–4.4)

CI: confidence interval; CT: chemotherapy; NR: not-reached; PFS: progression-free survival; RFS: recurrence-free survival.

## Data Availability

Raw data were generated at Instituto do Cancer do Estado de Sao Paulo. The derived data supporting the findings of this study are available from the corresponding author LGdF on request.
